# Utility of Cardiac Troponin-I in the Prediction of In-Hospital Mortality of Patients With COVID-19: A Retrospective Cohort Study in Central India

**DOI:** 10.7759/cureus.64327

**Published:** 2024-07-11

**Authors:** Himanika Paliwal, Nadia Noor Ali, Abhijit Ninghot, Azmat Kamal Ansari, Shabana Andleeb Ansari

**Affiliations:** 1 Department of Medicine and Surgery, Government Medical College Nagpur, Nagpur, IND; 2 Department of Biochemistry, Government Medical College Nagpur, Nagpur, IND; 3 Department of Biochemistry, Government Medical College Satara, Satara, IND; 4 Department of Biochemistry, Uttar Pradesh University of Medical Sciences, Etawah, IND; 5 Department of Pathology, Uttar Pradesh University of Medical Sciences, Etawah, IND

**Keywords:** cardiac troponin-i, mortality, prognosis, central india, covid-19

## Abstract

Laboratory tests have been used as prognostic markers in various diseases, especially those with infectious etiology, but the information on the role of biochemical parameters in the risk assessment of patients with COVID-19 is limited. We designed this retrospective cohort study to investigate the utility of troponin-I in predicting the in-hospital mortality of patients with COVID-19 admitted to our tertiary care hospital in central India. We strategically recorded the history, findings on physical examination, comorbid conditions, clinical diagnosis, results of the biochemical parameters, and adverse outcomes (in terms of survival or death) in order to assess the utility of troponin-I estimation done within the first 24 hours of admission in predicting the in-hospital mortality of patients with COVID-19. Appropriate statistical methods were used depending on the data generated to justify the aim of our study. P-values ​​less than 0.05 were considered significant. We observed a statistically higher (p=0.004) prevalence of mortality in the patients with higher troponin-I levels. We also observed a statistically significant association of other biochemical parameters with the mortality of these patients. Our study highlights the utility of troponin-I in predicting the in-hospital mortality of patients with COVID-19.

## Introduction

The International Federation of Clinical Chemistry and the Laboratory Medicine Task Force have effectively collected data on COVID-19 to facilitate specific recommendations for its diagnosis and treatment [1,2]. As these suggestions are mainly based on data from developed countries, research in resource-limited countries such as India is mandatory [3]. Since COVID-19 is known to have a variable clinical presentation that can range from asymptomatic infection to even death, early prediction of the disease severity can potentially impact the morbidity and mortality of this devastating disease [1,2].

Prognosis and early intervention are the cornerstones of the management of COVID-19 [4]. Early identification of individuals at a higher risk of mortality helps in the planning of appropriate interventions and thus increases the chances of survival [4-6]. Most of the models for prognosticating the adverse outcomes of COVID-19 are complicated because they are based on many variables (demographic variables, clinical profile, disease progression, etc.) [7]. Since healthcare resources in countries like ours can be severely compromised in such epidemics, a simple tool that can help in the better planning and use of the available resources would alleviate such situations [3,8].

In theory, the pathophysiological processes that are associated with the adverse outcomes should lead to molecular alteration that can be identified using the relevant biochemical parameters [9]. According to the Journal of the American College of Cardiology, elevations of the cardiac troponins are common in patients with COVID-19 [10]. The mechanisms proposed to explain this increase in troponins are stress cardiomyopathy, hypoxia due to the mismatch between supply and demand, acute coronary syndrome as a result of microvascular damage, systemic inflammatory response, and direct viral damage [10,11]. This association indicates that cardiac troponin levels can be used in the risk stratification, staging, and prognostication of patients with COVID-19 [12]. Therefore, we designed this study to evaluate the utility of troponin-I as a predictor of mortality in patients with COVID-19.

## Materials and methods

A laboratory-based retrospective observational cohort study was planned using the data from patients with COVID-19 admitted to our tertiary care center in central India between January and July 2021. After approval by the Institutional Ethics Committee of Government Medical College Nagpur (approval number: 3650EC/Pharmac/GMC/NGP), we retrospectively collected the data from the Hospital Information System and Laboratory Information System as well as the patients' files. We used the “Revised Guidelines for the Clinical Management of COVID-19” issued by the Government of India through the Directorate General of Health Services (DGHS), Ministry of Health and Welfare (EMR Division), for the diagnosis of COVID-19 patients [13]. These guidelines define COVID-19 patients as cases suspected to have COVID-19 infection with laboratory confirmation by either the real-time/TrueNat polymerase chain reaction (Molbio Diagnostics Pvt. Ltd., Goa, India) or rapid antigen test.

We included only the laboratory-confirmed COVID-19 patients whose troponin-I estimation was done within the first 24 hours of admission. The cases with a history of heart disease that were not managed according to the standard operating procedures (SOPs) for any reason (e.g., denial of consent, leave against medical advice, etc.) or without sufficient data (unavailability of the results of other biochemical parameters included in our study) for further analysis were excluded. The WBC and platelet counts were done on the Horiba hematology analyzer (Horiba, Ltd., Kyoto, Japan). Troponin-I and D-dimer assays were performed on an iChroma immunoassay analyzer (Boditech Med Inc., Gangwon-do, Korea). All other biochemical parameters included in our study were estimated using the XL 640 fully automatic (Erba Group, Czech Republic). The results of all parameters were validated via appropriate internal and external quality control protocols in our laboratory. The methodological details of all the parameters included in this study are summarized in Table 1.

**Table 1:** Methodological details of the biochemical parameters ALT: alanine transaminase, AST: aspartate transaminase, LDH: lactate dehydrogenase, hs-CRP: highly sensitive C-reactive protein, WBC: white blood cell, UV: ultraviolet, PLP: pyridoxal phosphate, ISE: ion-selective electrode, BCG: bromocresol green

Biochemical parameters	Analytical method	Reference range
Random plasma glucose	Hexokinase method	>140 mg/dl
Serum urea	Urease UV	17-43 mg/dl
Serum creatinine	Jaffe's kinetic-alkaline picrate	Males: 0.6-1.4 mg/dl, females: 0.6-1.2 mg/dl
Serum ALT	UV kinetic without PLP	<50 IU/L
Serum AST	UV kinetic without PLP	<50 IU/L
Serum sodium	ISE - indirect	136-146 mEq/L
Serum LDH	Kinetic UV	230-460 U/L
Serum ferritin	Electrochemiluminescence assay	10-29 ng/mL
Serum hs-CRP	Particle-enhanced immunoturbidimetry	>3 mg/L
D-dimer	Particle-enhanced immunoturbidimetry	0-0.5 mg/L
Serum total bilirubin	Diazonium salt	0.3-1.2 mg/dl
Serum albumin	BCG	3.5-5.2 gm/dl
WBC count	Electrical impedance	4.5-10.5 x10^9^/L
Platelet count	Electrical impedance	150-450 x10^9^/L

The cardiac troponin-I was assayed by fluorescence immunoassay. The normal troponin-I level was defined as equal to or less than 0.011 ng/mL (upper reference limit, i.e., 99th percentile) as described in the kits provided by the manufacturers. Troponin-I values greater than 0.011 ng/mL were considered elevated.

All the parameters were analyzed according to the SOPs of the respective sections of our laboratories. The patients were divided into two groups according to the outcomes (whether they died (deceased) or were discharged after the recovery (survivors)). They were further stratified into two groups depending on their cardiac troponin-I level: those with elevated (troponin-I level greater than 0.011 ng/mL) troponin-I levels or those with normal (troponin-I level equal to or less than 0.011 ng/mL) troponin-I levels. Patients were also classified according to the COVID-19 disease severity as per the “Revised Guidelines for the Clinical Management of COVID-19” issued by the Government of India through the Directorate General of Health Services (DGHS), Ministry of Health and Welfare (EMR Division) [13]. According to these guidelines, the COVID-19 cases were classified into mild (upper respiratory tract infection and fever without hypoxia or dyspnea), moderate (respiratory rate 24-30/min, dyspnea or spO2 between 90-93%), or severe (respiratory rate >30/min and shortness of breath with spO2 <90%) cases according to the severity of the disease [13].

The categorical variables were described as the frequencies and percentages, while the continuous variables were described as the means and standard deviations (SDs). The quantitative data were assessed for linearity using the Kolmogorov-Smirnov analysis, and the appropriate tests of statistical significance (Student's unpaired t-test or Mann-Whitney-U test) were used depending on the type of data. Using the MedCalc software (MedCalc Software Ltd., Ostend, Belgium), the means for the continuous variables were compared using the independent group p-values. The parameters with p-values less than 0.05 were considered statistically significant.

## Results

Among all the laboratory-confirmed COVID-19 patients hospitalized at our tertiary care center in central India between January and December 2021, 400 patients who had the results of troponin-I estimation done within 24 hours of hospitalization archived were randomly selected. Out of these, 236 cases were excluded due to a previous history of heart disease, non-compliance with the management SOPs for various reasons, and a lack of results for other biochemical parameters included in our study. Finally, 164 hospitalized laboratory-confirmed COVID-19 cases, both males and females, were selected.

Table 2 shows the baseline characteristics of the 164 selected laboratory-confirmed COVID-19 patients. Among these, 100 (61%) were males and 64 (39%) females, while 123 (75%) were survivors and 41 (25%) were deceased. After the categorization according to the disease severity, we observed that 79 (48.17%) patients had mild, 31 (18.90%) had moderate, and 54 (32.93%) had severe COVID-19 disease. Further, 144 (87.80%) had hypertension, 65 (39.60%) had diabetes mellitus (DM), three (1.83%) had asthma, three (1.83%) had chronic obstructive pulmonary disease (COPD), and five (3.05%) had chronic kidney disease (CKD).

**Table 2:** Baseline characteristics of the patients with COVID-19 DM: diabetes mellitus, COPD: chronic obstructive pulmonary disease, CKD: chronic kidney disease, COVID-19: coronavirus disease

Baseline characteristics		Frequency	Percentage (%)
Gender	Male	100	60.98%
	Female	64	39.02%
Life status	Survivors	123	75.0%
	Deceased	41	25.0%
COVID-19 disease severity	Mild	79	48.17%
	Moderate	31	18.90%
	Severe	54	32.93%
Hypertension	Present	144	87.80%
	Absent	20	12.20%
DM	Present	65	39.63%
	Absent	99	60.37%
Asthma	Present	3	1.83%
	Absent	161	98.17%
COPD	Present	3	1.83%
	Absent	161	98.17%
CKD	Present	5	3.05%
	Absent	159	96.95%

As shown in Table 3, elevated cardiac troponin-I levels were seen in 51 out of 164 patients (31.09%), while 113 out of 164 patients (68.90%) had normal troponin-I. The patients with elevated troponin-I were found to be older (65.20 ± 10.46 as compared to 61.07 ± 11.43 years, p=0.032), with a higher predominance of males (31 out of 51 (60.78%)) and a positive history of CKD (4 out of 51 (7.8%), p=0.015) as compared to patients with normal troponin-I. According to the severity of COVID-19, about 61 out of 113 (54%) cases with normal troponin-I had mild COVID-19 disease (p=0.006). Among the patients with elevated troponin-I, the most common comorbidity was hypertension (41 out of 51 (80.4%)), followed by DM (21 out of 51 (41.2%)) and CKD (4 out of 51 (7.8%)). We found that 23 out of 51 (45.1%) of the patients who had elevated troponin-I died, while 95 out of 113 (84%) of the patients with normal troponin-I survived (p<0.001).

**Table 3:** Comparison of clinical characteristics between groups of patients with COVID-19 based on their cardiac troponin-I level * statistically significant SD: standard deviation, DM: diabetes mellitus, COPD: chronic obstructive pulmonary disease, CKD: chronic kidney disease, COVID-19: coronavirus disease

Characteristics	Elevated troponin-I (n = 51)	Normal troponin-I (n = 113)	p-value
Age, mean (SD)	65.20 (10.46)	61.07 (11.43)	0.032*
Gender, female (%)	20 (39.2)	42 (37.2)	0.721
COVID-19 disease severity, mild (%)	14 (27.2)	61 (54.0)	0.006*
Hypertension (%)	41 (80.4)	100 (88.5)	0.247
DM (%)	21 (41.2)	43 (38.1)	0.624
Asthma (%)	1 (2.0)	2 (1.8)	0.918
COPD (%)	1 (2.0)	2 (1.8)	0.918
CKD (%)	4 (7.8)	1 (0.9)	0.015*
Life status, deceased (%)	23 (45.1)	18 (16.0)	<0.001*

As shown in Table 4, the patients with elevated cardiac troponin-I were observed to have biochemical and hematological abnormalities at the time of admission as well. We observed statistically significant increased levels of urea (p<0.001), creatinine (p<0.001), lactate dehydrogenase (LDH, p<0.001), highly sensitive C-reactive protein (hs-CRP, p=0.001), and WBC (p=0.007) and decreased levels of albumin (p=0.014) within the first 24 hours of admission of these patients.

**Table 4:** Comparison of biochemical parameters between groups of patients with COVID-19 based on their cardiac troponin-I level * statistically significant SD: standard deviation, ALT: alanine transaminase, AST: aspartate transaminase, LDH: lactate dehydrogenase, hs-CRP: highly sensitive C-reactive protein, WBC: white blood cells, COVID-19: coronavirus disease

Biochemical parameters	Elevated troponin-I (n=51)	Normal troponin-I (n=113)	p-value
Troponin-I, mean (SD)	0.075 (0.12)	0.008 (0.002)	<0.001*	
Glucose, mean (SD)	189.55 (109.14)	162.59 (77.36)	0.082	
Urea, mean (SD)	65.67 (41.53)	36.59 (22.37)	<0.001*	
Creatinine, mean (SD)	1.88 (1.38)	1.10 (0.44)	<0.001*	
ALT, mean (SD)	47.69 (64.12)	64.29 (97.46)	0.278	
AST, mean (SD)	81.76 (122.04)	69.67 (85.95)	0.493	
Sodium, mean (SD)	136.26 (8.79)	135.50 (5.54)	0.512	
LDH, mean (SD)	490.52 (282.25)	346.96 (157.14)	<0.001*	
Ferritin, mean (SD)	690.42 (514.11)	516.91 (468.36)	0.062	
hs-CRP, mean (SD)	50.39 (58.12)	27.09 (25.52)	0.001*	
D-dimer, mean (SD)	1.60 (2.36)	0.95 (1.86)	0.089	
Bilirubin, mean (SD)	0.61 (0.31)	0.54 (0.27)	0.179	
Albumin, mean (SD)	3.32 (0.39)	3.53 (0.48)	0.014*	
WBC, mean (SD)	9.07 (4.64)	7.16 (3.73)	0.007*	
Platelets, mean (SD)	185.72 (80.43)	196.08 (77.56)	0.453	

As shown in Table 5, 41 out of 164 (25%) patients died during hospitalization (deceased), while 123 out of 164 (75%) patients were discharged after recovery (survivors). The deceased were found to be older (66.15 ± 11.75 years as compared to 60.90 ± 11.2, p=0.010) and also had a positive history of CKD (4 out of 41 (9.8%), p=0.004). Only one out of 41 (2.4%) of patients categorized as having mild COVID-19 disease at the time of admission died after hospitalization (p<0.001). Further, two out of three (66.67%) patients with a history of COPD and four out of five (80%) patients suffering from CKD died, while all three patients suffering from asthma survived.

**Table 5:** Comparison of clinical characteristics between deceased and survivor groups * statistically significant at 95% confidence limit SD: standard deviation, COVID-19: coronavirus disease, DM: diabetes mellitus, COPD: chronic obstructive pulmonary disease, CKD: chronic kidney disease

Characteristics	Deceased (n=41)	Survivors (n=123)	p-value
Age, mean (SD)	66.15 (10.75)	60.90 (11.20)	0.010*
Gender, female (%)	16 (39.0)	48 (39.0)	1.00
COVID-19 disease severity, mild (%)	1 (2.4)	78 (63.4)	<0.001*
Hypertension (%)	31 (75.6)	113 (91.9)	0.006*
DM (%)	12 (29.3)	53 (43.1)	0.117
Asthma (%)	0 (0.0)	3 (2.4)	0.313
COPD (%)	2 (4.9)	1 (0.8)	0.093
CKD (%)	4 (9.8)	1 (0.8)	0.004*

As shown in Table 6, the deceased had statistically significant higher troponin-I (p=0.004), urea (p<0.001), creatinine (p<0.001), aspartate transaminase (AST, p=0.013), LDH (p<0.001), ferritin (p<0.001), hs-CRP (p<0.001), D-dimer levels (p=0.004), and WBC count (p<0.001) and lower albumin (p<0.001) and platelet count (p=0.040).

**Table 6:** Comparison of biochemical parameters between deceased and survivor groups * statistically significant at 95% confidence limit SD: standard deviation, ALT: alanine transaminase, AST: aspartate transaminase, LDH: lactate dehydrogenase, Hs-CPR: highly sensitive C-reactive protein, WBC: white blood cells

Laboratory paramaters	Deceased (n=41)	Survivors (n=123)	p-value
Troponin-I, mean (SD)	0.058 (0.13)	0.019 (0.03)	0.004*
Glucose, mean (SD)	183.76 (102.17)	165.29 (83.70)	0.259
Urea, mean (SD)	73.41 (44.34)	35.74 (19.36)	<0.001*
Creatinine, mean (SD)	2.03 (1.37)	1.11 (0.53)	<0.001*
ALT, mean (SD)	76.12 (126.07)	52.61 (69.45)	0.138
AST, mean (SD)	106.30 (141.59)	61.46 (72.87)	0.013*
Sodium, mean (SD)	136.70 (7.50)	135.32 (6.41)	0.264
LDH, mean (SD)	595.60 (290.06)	339.28 (146.80)	<0.001*
Ferritin, mean (SD)	908.33 (486.83)	469.36 (445.24)	<0.001*
Hs-CPR, mean (SD)	59.33 (64.86)	25.58 (20.37)	<0.001*
D-dimer, mean (SD)	2.04 (2.85)	0.87 (1.61)	0.004*
Bilirubin, mean (SD)	0.61 (0.35)	0.54 (0.26)	0.220
Albumin, mean (SD)	3.16 (0.42)	3.57 (0.42)	<0.001*
WBC, mean (SD)	9.85 (5.57)	7.04 (3.18)	<0.001*
Platelets, mean (SD)	172.80 (78.81)	202.58 (78.92)	0.040*

Table 7 shows that the cardiac troponin-I has a positive predictive value of 71.91% with a 95% confidence interval (60.41% to 81.11%) and a negative predictive value of 64.10% with a 95% confidence interval (57.54% to 70.17%). The sensitivity and specificity were 46.94% (32.53% to 61.73%) and 83.78% (75.59% to 90.10%), respectively. This shows that 71.91% is the probability that a patient with elevated cardiac troponin-I levels within the first 24 hours of admission will die during treatment, while 64.10% is the probability that a patient with normal cardiac troponin-I will be discharged after the recovery. We observed 46.93% disease (mortality) prevalence, which indicates that 46.93% is the percentage of patients who had elevated troponin-I and died.

**Table 7:** Cardiac troponin-I level as a predictor of mortality in patients with COVID-19 COVID-19: coronavirus disease

Statistic	Value	95% confidence interval
Sensitivity	46.94%	32.53-61.73%
Specificity	83.78%	75.59-90.10%
Positive likelihood ratio	2.89	1.73-4.85
Negative likelihood ratio	0.63	0.48-0.83
Disease (mortality) prevalence	46.93% (mortality)	
Positive predictive value	71.91%	60.41-81.11%
Negative predictive value	64.10%	57.54-70.17%
Accuracy	66.49%	58.61-73.75%

## Discussion

Our study demonstrated statistically significant associations between elevated troponin-I levels and the disease severity as well as the in-hospital mortality of hospitalized COVID-19 patients. According to our findings, the troponin-I estimation within the first 24 hours of admission to the hospital can be used for the screening and risk assessment of patients with COVID-19. Troponin-I is a cardiac-specific, highly sensitive marker of myocardial damage. Although evidence of a COVID-19-associated increase in cardiac troponin levels is emerging, the exact mechanism of this phenomenon is still under research [10-12,14]. One of the mechanisms proposed behind the cardiac troponin elevation is the cytokine storm [15]. It is hypothesized to cause myocyte stress and injury, fibroblast activation, inflammatory modulation, and extracellular matrix remodeling [15-16]. A significant elevation in the inflammatory markers such as hs-CRP (p<0.001) and ferritin (p<0.001) in the deceased, as observed in our study, also points toward the cytokine storm as the mechanism for severe COVID-19 infection and the resultant adverse outcomes. However, the inclusion of patients with renal dysfunction may result in a confounding elevation in troponin levels [10,12]. Studies of other confounding factors are required to validate these findings.

The deceased were observed to have statistically significant higher levels of troponin-I (p=0.004), urea (p<0.001), creatinine (p<0.001), AST (p=0.013), LDH (p<0.001), ferritin (p<0.001), hs-CRP (p<0.001), D-dimer levels (p=0.004), and WBC count (p<0.001) and lower levels of albumin (p<0.001) and platelet count (p=0.040) within the 24 hours of hospitalization. Several studies have reported the association of these parameters with mortality in COVID-19 [3,6,9,15-17]. Other biochemical markers (glucose, ALT, sodium, and bilirubin) did not differ significantly between the groups of survivors and deceased (p>0.05). Few studies have proposed these parameters as predictors of mortality, but in the present study, these parameters failed as prognostic markers of mortality [17].

We observed statistically significant increased levels of urea (p<0.001), creatinine (p<0.001), LDH (p<0.001), hs-CRP (p=0.001), and WBC (p=0.007) and decreased levels of albumin (p=0.014) in patients with elevated troponin-I levels. This observation points toward the predisposition of patients with high troponin levels to more severe COVID-19 disease manifestations and subsequent multi-organ damage resulting in biochemical alterations, as proposed by previous studies [2,3,6,9-12,15-17]. This finding also conciliates with the findings of several studies that suggest a correlation between CKD and increased levels of cardiac troponins [10,12].

In our study, COVID-19 patients showed distinct clinical, demographic, and laboratory features in the first 24 hours of their hospitalization. It was observed that males and those with higher ages were prone to mortality. Previous studies have also reported similar observations. Research showed that this could be due to the effect of gender and age on the angiotensin-converting enzyme 2 (ACE2) receptor expression [18-19]. ACE2, which acts as a functional receptor for COVID-19, has been found to be overexpressed in males. This may be due to the androgen-mediated expression of ACE2 [18]. Aging causes immunosenescence (a gradual decline in immune function) and inflammaging (a chronic increase in systemic inflammation), along with the increased prevalence of other comorbidities, making them more vulnerable to severe infections and death [18-20].

With regard to comorbid conditions, mortality was found to be significantly higher in cases with a positive history of CKD. Patients with a history of CKD showed elevated troponin-I levels and were more prone to adverse outcomes. Studies have reported similar observations, but more research on the pathophysiology of this observation is required [18]. CKD not only affects immune homeostasis but also delays the clearance of cytokines, which leads to a prolonged inflammatory state [21]. This explains why patients with CKD had a higher mortality rate in our study. To our surprise, the prevalence of hypertension was higher among the survivors in the present study. This finding conflicts with earlier studies [6-9]. As the details required for classification (grades according to systolic and diastolic blood pressure (BP)) of hypertensive patients enrolled in our study are not available, no conclusions can be drawn from this unusual finding.

Based on the results of our study, we suggest that biochemical parameters, especially troponin-I (as it correlates well with disease severity as well as mortality), can be very useful in prognostication. Patients with COVID-19 who are at higher risk can be handled effectively with the aid of such prognostic markers [22]. The utilization of the existing resources can be improved with prompt prognostication and timely interventions with the help of such screening strategies [22]. The biggest challenge with the prediction of diseases like COVID-19 is the prediction of the requirement and planning of adequate ventilatory support, as the condition of these patients is highly dynamic [23]. The idea of using cardiac troponin for risk stratification in order to facilitate more comprehensive monitoring is theoretically very promising. The findings of our study point towards the need for validation of this assumption with further studies with a larger sample size and better design. Consideration of additional variables in the prognostication of adverse outcomes in COVID-19 would be more scientific, but the present study aimed to assess the utility of troponin-I as a simple screening test for the identification of COVID-19 patients with higher risk.

The conduct of this study at a renowned tertiary care hospital in central India, known for its state-of-the-art facilities and quality care, is the main strength of our study. Some of the other advantages include its appropriate inclusion criteria, scientific research methods, evidence-based analytical methods for all the parameters, and management of all patients with uniform SOPs. Moreover, as the study focused on the utility of a single biochemical parameter, i.e., troponin-I, that is easily accessible in the majority of critical care units, it may provide a practical tool for prognostication in COVID-19. During the COVID-19 pandemic, we have learned that it is not feasible to use complex predictive models in resource-limited setups. In such circumstances, simple screening tests to allow for easier prognostication can be really helpful. The major drawback of our study is its retrospective design and small sample size. Studies with a larger sample size and a special emphasis on the validation of these findings are required. Another drawback is that although the troponin-I estimation was done within the first 24 hours of hospitalization for all the patients, the further categorization of study subjects was not time-specific. Further, the inclusion of patients with renal or other organ dysfunction and incomplete data on vitals like BP, peripheral oxygen saturation, SPO2, etc. adds to the weakness of our study. Moreover, the variance of the disease progression at the time of sample collection for these biochemical parameters was not taken into consideration.

## Conclusions

Troponin-I can be used for early prediction of the disease severity as well as mortality of COVID-19 patients. We propose that early prognostication with the help of monitoring of troponin-I and other biochemical parameters can facilitate timely intervention along with better resource management and thus better patient outcomes, especially in resource-limited setups.
